# Collagen Biosynthesis and Its Molecular Ensemble: What Remains Unexplored

**DOI:** 10.1021/acs.biochem.5c00261

**Published:** 2025-07-10

**Authors:** Yoshihiro Ishikawa

**Affiliations:** Department of Ophthalmology, University of California San Francisco, School of Medicine, San Francisco, California 94158, United States

## Abstract

Collagen embodies an intriguing paradox in protein biology. Despite being one of the most abundant protein superfamilies in vertebrates and having a seemingly simple structural organization, its biosynthesis is anything but straightforward. This apparent simplicity masks a complex and often contradictory biosynthetic landscape that poses significant challenges, particularly for newcomers to the field. Rather than following a linear or uniform pathway, collagen biosynthesis involves a coordinated series of tightly regulated steps, cotranslational post-translational modifications (PTMs), chain selection and registration, triple helix formation, and secretion, orchestrated by a specialized machinery, collectively termed the collagen molecular ensemble. This ensemble must overcome unconventional paradigms in protein biogenesis, rife with exceptions and unresolved questions. In this perspective, I examine underexplored aspects of the collagen biosynthetic machinery, spotlighting challenges in decoding the regulatory logic of PTMs, the spatial dynamics of trimer assembly, the functional consequences of chain registration, and the type-specific routes of secretion. By charting these uncertainties, I aim to challenge prevailing assumptions and invite interdisciplinary insight to help unravel the remaining mysteries of collagen biosynthesis.

## Graphical abstract

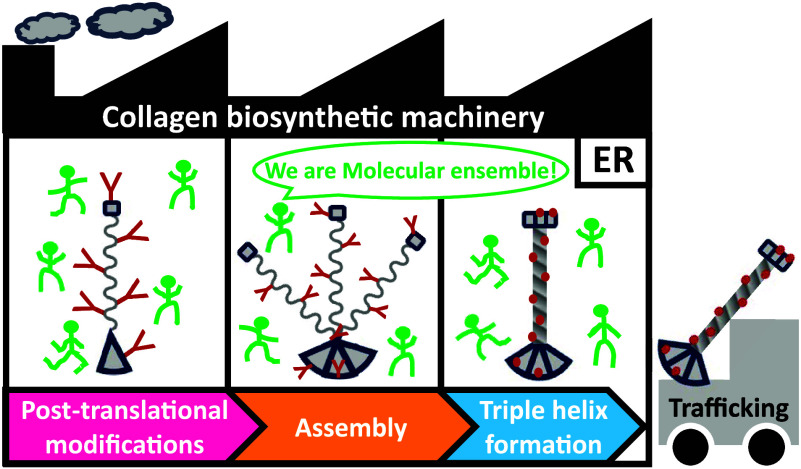

## Introduction – Collagen and Its Biosynthesis at a Glance

Collagen, which accounts for one of the most abundant proteins in vertebrates,^1^ forms a superfamily composed of 28 different types of trimeric protein complexes encoded by 44 genes in humans.^2^ Beyond canonical collagens, an additional type of molecule, exemplified by adiponectin^3^ and C1q,^4^ possesses characteristic collagenous domains (Gly-X-Y repeats). Given their collagen-like architecture, often referred to as collagen-like proteins, I acknowledge that we consider them within a broader classification related to collagen biology. This protein family plays fundamental roles, from maintaining skeletal and tissue architecture to serving as signaling platforms through fibril- or sheet-like structures. The elongated Gly-X-Y repeat-containing region, known as the collagenous domain (with up to 338 repeats in collagen I), is flanked by amino- (N-) and carboxyl- (C−) terminal noncollagenous (NC) domains. Each collagen molecule is composed of three polypeptide chains that wind together into a rope-like structure known as the triple helix, the defining hallmark of collagen.^5^ At first glance, this repetitive Gly-X-Y motif suggests a straightforward biosynthetic pathway and suitability for large-scale production. However, this apparent simplicity often masks a complex and exceptional protein biosynthesis that has historically and practically made collagen research challenging. In fact, collagen and its biosynthesis constitute a more intricate paradigm compared with protein folding in general. To manage this challenge, cells employ a sophisticated biosynthetic machinery involving over 20 enzymes and chaperones within the endoplasmic reticulum (ER), collectively referred to here as the collagen molecular ensemble.^6^ This ensemble orchestrates four major steps: cotranslational post-translational modifications (PTMs), assembly, triple helix formation, and ER-to-Golgi trafficking (Figure 1). Each of these steps includes exceptions and unresolved mechanisms that need to be fully understood. In this perspective, I highlight less explored territories within the collagen molecular ensemble. By mapping these uncertainties, I aim to challenge prevailing assumptions and invite diverse expertise to unravel the remaining mysteries of collagen biosynthesis in the ER. I anticipate that these efforts will have broad implications for collagen-based applications in basic protein science, disease treatment, tissue engineering, precision medicine, drug delivery, and biomedical device development.

**Figure 1:**
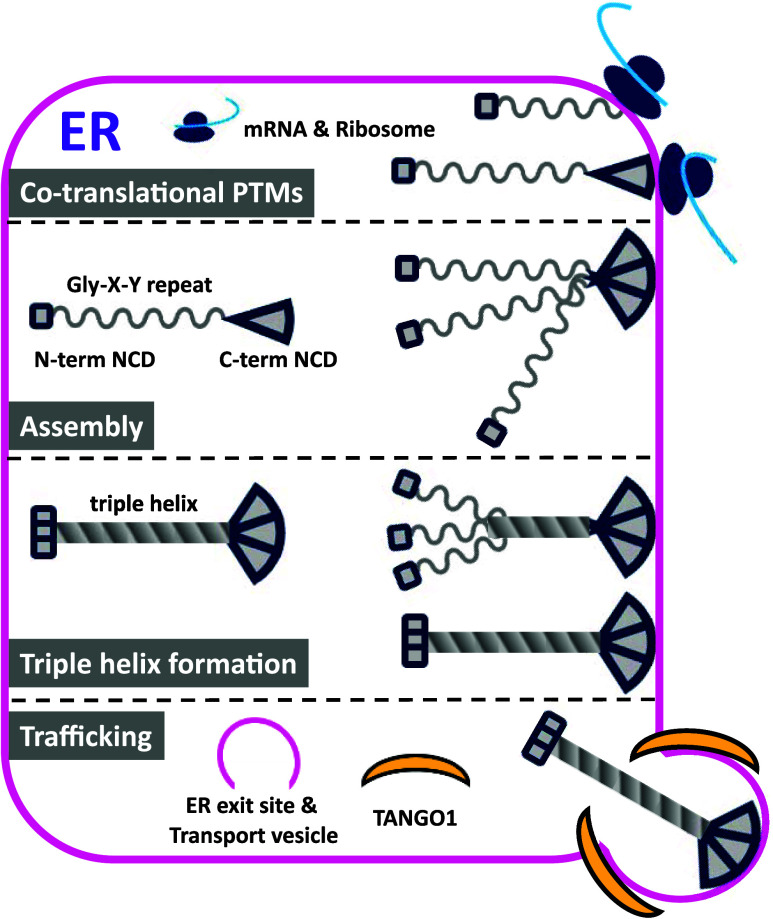
Schematic overview of the four major steps in collagen biosynthesis within the ER.

Within the ER, collagen Gly-X-Y polypeptides undergo cotranslational post-translational modifications (PTMs) such as hydroxylation and glycosylation. The modified chains assemble into trimers via the C-terminal noncollagenous domain (NCD). Trimeric complexes initiate triple helix formation from the C- to N-terminus. Properly folded collagen is transported toward the secretory pathway mediated by TANGO1. This schematic illustrates stepwise progression, cotranslational PTMs, assembly, triple helix formation, and trafficking, driven by the collagen biosynthetic machinery.

## Post-Translational Modifications (PTMS): What Causes Their Differences among Collagen Types?

Newly synthesized collagen polypeptides undergo extensive PTMs within the ER lumen. Some of their X and Y position proline (Pro) and Y position lysine (Lys) residues in Gly-X-Y triplets are hydroxylated by collagen modifying enzymes prolyl 3-hydroxylases,^7^ prolyl 4-hydroxylases,^8^ and lysyl hydroxylases,^9^ respectively. By a distinct set of collagen glycosyltransferases, hydroxylated lysine (hydroxylysine, Hyl) is further modified to collagen-specific glycosylated Hyl, such as galactosyl-Hyl and glucosylgalactosyl-Hyl.^10^ In these PTMs, 4-hydroxyproline consistently appears with high abundance across the collagen superfamily.^11^ Conversely, 3-hydroxyproline and lysine modifications display substantial variability across collagen types.^12,13^ This variability suggests the existence of specific regulatory rules or modulators that remain unidentified. It is intriguing to note the other PTM, *N*-linked glycosylation, in collagen Gly-X-Y repeats. In the ER, chaperones recognize this glycosylation as a quality control marker to facilitate proper protein folding.^14,15^ Since this modification requires an Asn-X-Ser/Thr motif, it can only occur within the Gly-X-Y sequence as Gly-X-Asn-Gly-Ser/Thr-Y-Gly. While the role of *N*-glycosylation in the C-terminal NC domains of fibrillar collagens is well studied,^16^ its precise function within the Gly-X-Y repeats remains undefined. Beyond physiological conditions, studies of human pathology, particularly osteogenesis imperfecta, have revealed a practical rule of how this disorder slows down collagen triple helix formation, leading to excess lysine PTMs.^17–19^ This phenomenon aligns with collagen triple helix formation, significantly differing from the typical folding mechanisms of multidomain proteins, which fold cotranslationally. The collagen triple helix initiates at the C-terminal NC domain and proceeds toward the N-terminus, indicating that helix formation is deferred until polypeptide translation and subsequent PTMs are complete. This unique folding mechanism could provide the necessary “time” for the appropriate amount of PTMs to occur, as these modifications cannot be added once the triple helix has formed. Therefore, deviations in the rate of folding, either faster or slower, can alter the extent of lysine modifications, underscoring a tight association between triple helix formation rates and collagen PTMs. Interestingly, even under the same principle of physiological collagen triple helix formation, the spectrum of lysine PTMs differs between collagens. In fibrillar collagens, types II, V, and XI exhibit much higher levels of lysine PTMs than types I and III.^12,20–23^ A plausible explanation for this difference involves the activity of peptidylprolyl cis/trans isomerases (PPIases). Proline residues frequently occupy positions X and Y within collagen Gly-X-Y repeats. Since all proline peptide bonds are fixed in the *trans* conformation in the collagen triple helix, proline residues must isomerize from the *cis* to the *trans* state.^24^ This isomerization is considered a rate-limiting step, and this biochemical event is facilitated by the ER-resident PPIases. Of the seven PPIases present in the ER, six have been biochemically characterized regarding their involvement in collagen triple helix formation (Table 1). Their activities vary depending on the amino acid preceding the proline residue (the X position in the X-P sequence) and the presence of proline PTMs.^25^ While PPIases relevant to collagens I and III have been well characterized, specificities among the seven ER-resident PPIases for the 28 collagen types remain unclear. Given that amino acid composition is critical for the activity of these ER PPIases, reevaluating the frequency and pattern of Gly-X-Y repeats might unveil new biological codes and/or messages embedded in different collagens.

**Table 1:**
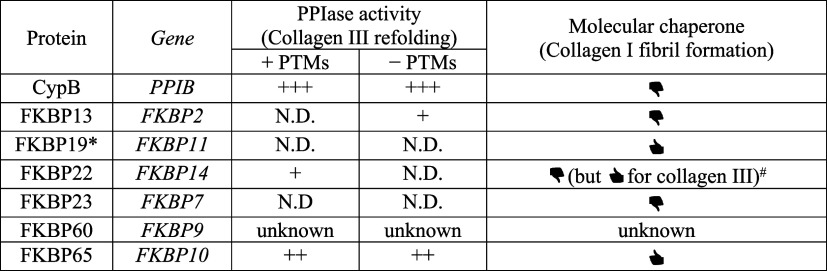
Summary of *In Vitro* Studies Characterizing ER-Resident PPIases with Collagen Substrates

* Human FKBP19 is the FKBP domain of FKBP19 in this table. +++, + +, +, and N.D. indicate strong, moderate, weak, and not detected, respectively. Black solid thumbs up and black solid thumbs down indicate inhibition and no inhibition of collagen I fibril formation. #: FKBP22 inhibits fibril formation of collagen III rather than collagen I. This table was compiled using data from the following refs 25–100 .

## Collagen Trimer Assembly: Chains Encounter into the Right Trio as Efficiency Mechanisms in the Crowded ER Environment

The assembly of three collagen chains into a triple helix is a remarkable feat, especially considering the crowded protein biogenesis environment of the ER lumen. This process is attributed to the presence of specialized trimerization domains, mostly located at the C-terminal ends of the procollagen chains.^28^ These domains facilitate the recognition and assembly of three collagen chains, ensuring the correct chain combination is essential for triple helix formation. The trimerization of collagen I chains is suggested to occur in close proximity to the ER membrane, mediated by the membrane protein calnexin through the N-linked glycosylation in the C-terminal trimerization domain.^16,29^ This model appears highly plausible as the tethering of the chains to the ER membrane allows for two-dimensional lateral movement along its surface. This significantly increases the likelihood of three distinct chains encountering each other and initiating their trimerization compared to a trimolecular reaction occurring freely within the three-dimensional space of the ER lumen. Given their structural similarities, other fibrillar collagens likely follow the same principle. Although the collagen IV trimerization domains are well characterized, they lack the site anchoring N-linked glycosylation, and their trimeric form is an unstable complex.^30^ Therefore, it remains unknown whether other collagen types similarly utilize the ER membrane for efficient assembly or predominantly assemble within the ER lumen. In summary, identifying facilitators or regulatory factors responsible for ensuring the correct trimer assembly in the ER remains an important unresolved challenge.

It is important to note that many studies assessing collagen expression focus exclusively on single-chain mRNA expression. This approach is inherently limited, as it does not inform us whether a single chain effectively encounters partner chains to form a proper trimer, except in the case of homotrimeric collagens composed of identical chains. As described earlier regarding collagen PTMs, the efficiency of chain selection, assembly, and subsequent triple helical formation influences the accessibility of the collagen modifying enzymes. Future research should clarify the kinetic relationships among translation, trimer assembly, and triple helix formation across different collagen types. This could reveal general principles governing the extent of PTMs.

## Faces of Collagen: Surface Pattern Diversity through Chain Registration

The surface of the collagen triple helix utilizes Gly-X-Y triplets, maximizing the reactivity of amino acids on the surface through the exposure of the X and Y amino acids since glycine is located at the core of the triple helix.^31^ This elegant structural arrangement enables collagen to perform its essential functions in the extracellular matrix (ECM), including interactions with other ECM proteins, signaling molecules, and cell surface receptors. Importantly, except for homotrimeric collagens, the collagen surface theoretically differentiates depending on the registration of the three chains. In other words, when three distinct collagen chains assemble into a triple helix, each chain adopts a specific position, leading, middle, or trailing, along the helical winding.^32,33^ These positional assignments define unique surface topographies. Figure 2 illustrates this concept: from a mathematical perspective, three artificial collagen chains (Figure 2A) have six possible distinct chain registrations (Figure 2B). A magnified view of a specific block (Figure 2C) reveals apparently similar but mechanistically distinct patterns between each registration (Figure 2D). The electrostatic interaction on the surface of the triple helix (a white line in Figure 2D) is well discussed in this latest report.^34^

**Figure 2:**
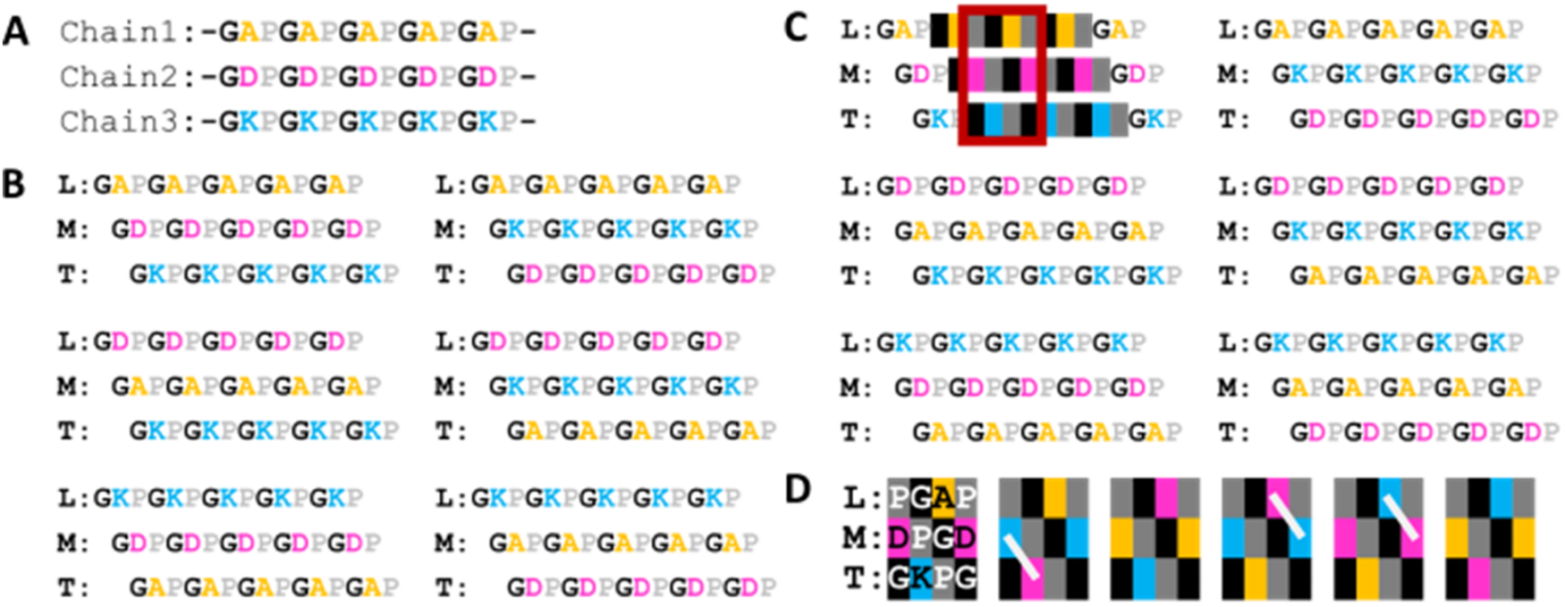
Collagen chain registration and surface heterogeneity. (A) Schematic of three artificial collagen chains composed of simple Gly-X-Y repeats. Glycine (G) is shown in black, and the X and Y positions are color-coded by residue type to highlight positional differences. (B) Six theoretically possible chain registrations for the three artificial collagen chains shown in panel (A). L, M, and T indicate Leading, Middle, and Trailing positions within the triple helix, respectively. (C) Selected region is highlighted with color blocks, and the magnified segment is boxed in red. (D) Schematic representation of the surface patterns generated by the six different chain registrations shown in panel (B), focusing on the segment annotated in panel (C). White line indicates the potential formation of an electrostatic interaction between the lysine (K) and aspartic acid (D) residues.

Collectively, even with simple artificial sequences, these differences hint at the potential complexity and biological significance of surface heterogeneity in native heterotrimeric collagens. Here, I emphasize that this simplified model does not account for any PTMs. Therefore, when discussing collagen in its natural context, its surface heterogeneity should be considered, with PTMs as an additional parameter. While PTMs are often discussed in the context of protein stability and protein–protein interactions, their potential role in chain registration has not been thoroughly explored. For example, the C-terminal prolyl 3-hydroxylation at position P986 (counted from the first Gly-X-Y triplet) in the α1 chain of collagen I plays a critical role. Loss of this modification leads to osteogenesis imperfecta^18,19^ and has been proposed to influence interactions between collagen and ECM proteins.^35^ However, knock-in mice carrying a Col1a1 P986A substitution, which disrupts the native Gly-X-Y triplet but prevents prolyl 3-hydroxylation, surprisingly does not exhibit recessive bone dysplasia, although collagen cross-linking and structural organization are affected.^36^ Given that triple helix formation proceeds from the C- to N-terminus, it is an intriguing possibility that this prolyl 3-hydroxylation modulates chain registration. In addition to PTMs, while fibrillar collagens maintain single chain registration due to continuous linear Gly-X-Y repeats, nonfibrillar collagens, such as collagen IV, contain interruptions.^37^ These interruptions result in multiple distinct triple-helical segments within the same molecule. It remains an open question whether chain registration is maintained or differs among each segment. Because these surface features may govern specific protein–protein interactions, more attention should be paid to the concept of *collagen surface probabilities*, a structural variable with functional consequences that is currently underappreciated.

## Collagen Trafficking: Do Collagen Type-Dependent Routes Exist from the ER to the ECM?

Protein trafficking, particularly of extralarge molecules such as collagens, is an area of active research focused on understanding their specialized secretion mechanism. In this context, a seminal paper published in 2009 ^38^ significantly advanced the field. The discovery of TANGO1 (Transport and Golgi Organization 1) revealed how collagens are packed into secretory vesicles. Since then, many studies have explored TANGO1-mediated collagen trafficking. Currently, various transport routes from the ER to the ECM via the Golgi apparatus have been proposed for collagens. These include a route utilizing conventional COPII vesicles,^39^ generating mega vesicles,^40,41^ and opening elongated tube^42^ and tunnel structures^43,44^ to the Golgi. Furthermore, evidence suggests that the selection of these routes depends on collagen types.^45–49^ This underscores the necessity of considering structural and molecular diversity among collagen types. A key consideration within this field is the careful distinction of the collagen types. It is time to systematically examine the correlation between secretory pathways and collagen types. Moreover, the choice of the secretory pathway may be influenced by environmental factors, such as cell types, physiological state,^39^ stress conditions,^50^ or pathological conditions.^51,52^ The advances in our understanding of collagen trafficking have been driven by technological breakthroughs, particularly improvements in imaging quality and manipulation of collagen genes with fluorophores. Indeed, fluorophore-tagged collagen molecules in both cellular and animal models have become increasingly prevalent.^45,53–60^ While observing the growing interest in collagen trafficking is fascinating, fluorescence-based collagen studies require careful validation to ensure that the tagged collagen maintains proper triple-helical formation. In my view, a full understanding of TANGO1-mediated collagen trafficking will require complementary biochemical and biophysical investigations of collagen molecules themselves in addition to current imaging-based approaches. A synergistic collaboration between cell biologists, protein-imaging experts, and collagen biochemists/biophysicists could unlock new avenues for elucidating fundamental collagen trafficking mechanisms as well as for discovering novel therapeutic targets. In closing this section, I revisited overlooked findings that could inform future discussions. Two studies have proposed a compelling collagen trafficking model: one involving a TANGO1 knockout (KO) mouse model^61^ and another examining the interaction between TANGO1 and HSP47.^62^ TANGO1 KO mice exhibited impaired secretion of collagens I/II/III/IX, IV and VII, in chondrocytes, endothelial and mural cells, and mouse embryonic fibroblasts, respectively,^61^ suggesting TANGO1 is globally involved in collagens secretion. The essential collagen molecular chaperone HSP47 interacts with the SH3 (Src-homology 3) domain at the N-terminal edge of the long arm of TANGO1 in the ER, indicating that HSP47 acts as an anchoring molecule between collagens and TANGO1.^62^ This model seemingly provides an elegant explanation of the prevailing reasons and how TANGO1 facilitates collagen trafficking. However, as described above, reality is more complex. It is important to note that the 2009 paper that redirected the field has already demonstrated this complexity: TANGO1 KO fibroblast cells showed impaired secretion of collagen VII but maintained normal secretion of collagen I,^38^ suggesting that the contribution of TANGO1 to collagen I secretion may vary between tissues and cell types, such as between chondrocytes and fibroblasts. In future research on TANGO1-associated collagen trafficking, attention should be paid to (1) specifying the rule governing collagen types and trafficking routes, (2) assessing whether the quality of secreted collagens, such as their PTMs, affects trafficking routes, and (3) clarifying if the intracellular environmental parameters affect their trafficking routes. A comprehensive overview of TANGO1 and related proteins is well documented in this latest report.^63^ It is essential to recognize the importance of post-Golgi trafficking in collagen biology.^64^ This step is crucial for fibrillar collagen maturation, including the proteolytic processing of NC domains^65^ and the role of the fibripositor as a seed for collagen fibril growth.^66^ A notable and unresolved question is whether the ER-to-ECM transport routes involving the Golgi apparatus operate in a coordinated or compartmentalized manner. Future work should carefully examine the interplay between the ER-to-Golgi pathway (discussed above), Golgi-to-plasma membrane trafficking,^67^ and endocytic recycling routes,^68^ particularly in the context of collagen type-specific differences and large-scale processes such as fibrillogenesis.

## Take Home Message and Future Directions

Collagen biosynthesis is often described as a complex process, which is true; however, this description can serve both as a beneficial agenda and a convenient excuse for researchers. Ideally, our goal should be to unravel these complexities and present a clear blueprint of the process. I anticipate that the study of collagen biosynthesis continues to be an open and vibrant field enriched by the active exchange of ideas between researchers. There remain many aspects to explore in collagen biosynthesis, such as ribosome quality control, droplet formation, and environmental factors in the ER. This brief article can only scratch the surface; I hope it will inspire ideas, perhaps by initiating a ripple effect, like a stone thrown into an ocean.
